# The Relationship Between Interferon Status and HPV Infection in Patients with Oral Epithelial Dysplasia: An Observational Study

**DOI:** 10.3390/clinpract16100179

**Published:** 2026-09-28

**Authors:** Guzel Khisamieva, Diana Sologova, Oxana Svitich, Ekaterina Diachkova, Victoria Morozova, Anna Maltseva, Rashid Aliyev, Mikhail Stepanov

**Affiliations:** 1 Department of Oral Surgery of the Institute of Dentistry, I.M. Sechenov First Moscow State Medical University (Sechenov University), 119048 Moscow, Russia; sologova.diana@yandex.ru (D.S.); secu2003@mail.ru (E.D.); morozova_v_v@staff.sechenov.ru (V.M.); rashid_1311@icloud.com (R.A.); stepanov_m_a@staff.sechenov.ru (M.S.); 2 Department of Microbiology, Virology, and Immunology, I.M. Sechenov First Moscow State Medical University (Sechenov University), 125009 Moscow, Russia; svitich_o_a@staff.sechenov.ru; 3 I.I. Mechnikov Research Institute of Vaccines and Sera, 105064 Moscow, Russia; 4 Department of Orthodontics and Prevention of Dental Diseases of the Institute of Dentistry, I.M. Sechenov First Moscow State Medical University (Sechenov University), 119048 Moscow, Russia; maltseva_a_g@staff.sechenov.ru

**Keywords:** interferon status, oral potentially malignant diseases, epithelial dysplasia, oral mucosa, human papillomavirus

## Abstract

**Background/Objectives**: Oral potentially malignant diseases (OPMDs) with signs of epithelial dysplasia are characterized by a high risk of malignancy. However, there are still no algorithms for their early diagnosis and prediction, due to the many possible predisposing factors, including human papillomavirus (HPV) infection and decreased immunity. There is a need to assess the relationship between the manifestations of oral epithelial dysplasia (OED), HPV invasion in the oral cavity, and interferon status to improve the identification and characterization of patients with different degrees of dysplasia. **Methods**: This study was designed and reported with reference to the STROBE checklist. The study aimed to assess the association between interferon status and HPV invasion in patients with OED. As per the inclusion and exclusion criteria, this observational study included 180 patients with histologically confirmed oral epithelial dysplasia associated with leukoplakia or oral lichen planus. Interferon (IFN) status was assessed by measuring serum interferon activity, spontaneous interferon production, IFN-α/β, and IFN-γ activity in peripheral blood. HPV DNA was detected and genotyped in biopsy specimens using polymerase chain reaction. **Results**: HPV-positive patients demonstrated significantly lower IFN-α/β and IFN-γ activity compared with HPV-negative patients (*p* < 0.001). Severe OED was more frequently observed in HPV-positive individuals, whereas mild dysplasia predominated among HPV-negative patients (*p* < 0.05). Interferon activity progressively decreased with increasing dysplasia severity, with the lowest IFN-α/β and IFN-γ activity detected in patients with severe dysplasia (*p* < 0.001 and *p* < 0.05, respectively). A significant positive correlation between IFN-α/β and IFN-γ activity was identified in patients with moderate dysplasia (*p* = 0.003). Multivariate binary logistic regression analysis demonstrated that HPV positivity (adjusted OR = 4.62, 95% CI: 2.18–9.79; *p* < 0.001) and reduced IFN-α/β activity (adjusted OR = 1.34 per 10-unit decrease, 95% CI: 1.14–1.58; *p* < 0.001) were independently associated with severe dysplasia. The multivariable model demonstrated acceptable discriminatory ability for identifying patients with severe dysplasia (AUC = 0.736), with a sensitivity of 89.4% and a specificity of 56.1%. **Conclusions**: The findings suggest that HPV-associated OED is linked to reduced interferon status, particularly lower IFN-α/β and IFN-γ activity in more severe dysplastic lesions. Combined assessment of interferon status and HPV detection may help identify patients at higher risk of severe OED.

## 1. Introduction

The clinical importance of choosing methods for the examination and treatment of patients with oral potentially malignant diseases (OPMDs) associated with the human papillomavirus (HPV) is explained, on the one hand, by their prevalence and, on the other hand, by the lack of an effective diagnostic algorithm for this pathology that has been widely implemented.

OPMD is a group of conditions with a risk of malignancy [1,2]. Oral epithelial dysplasia (OED) is the most common pathohistological sign of this group of diseases [3]. It is known that OED is a pathoanatomical diagnosis that is made on the basis of observed cytological and architectural changes in tissue [2,3]. Thus, this term is applied to a lesion in which the entire thickness of the epithelium or part of it is replaced by cells showing varying degrees of cellular atypia [3,4].

OED increases the likelihood of transformation of OPMD into oral squamous cell carcinoma (OSCC). Conditions closely associated with the development of dysplasia and OSCC include such diagnoses as verrucous leukoplakia, erythroplakia, submucous fibrosis, and hyperkeratotic and erosive ulcerative forms of oral lichen planus (OLP) [1,2,3]. A number of authors have found that the frequency of malignant transformation of OPMD with diagnosed OED is from 5% to 36%, depending on its severity [5]. According to the current (5th edition, 2022) classification of the World Health Organization (WHO), epithelial dysplasia is graded using a three-tiered system (mild, moderate, and severe), with carcinoma in situ subsumed under severe dysplasia [6]. An alternative binary system (low-grade/high-grade), based on architectural and cytological criteria proposed by Kujan et al., has also been validated and is increasingly used in clinical practice [7]. The diagnosis is based on an assessment of various architectural and cytological abnormalities detected by light microscopy.

The etiology of most precancerous diseases of oral mucosa with signs of OED is not definitively clear. It is known that there are predisposing factors contributing to their development. According to many studies, one of the main factors associated with the risk of developing OPMD and OSCC is the human papillomavirus [1,3].

HPV is a DNA-containing virus of the Papillomaviridae family, which is tropic to the epithelium of the skin and mucous membranes. To date, more than 220 HPV types have been identified, which are divided into high- and low-carcinogenic risk virus types [8]. A study by Tilakaratne WM et al. (2019) indicates that the overall prevalence of HPV with high carcinogenic risk in oral and oropharyngeal dysplasia is 24.5% [3]. According to statistics, dysplasia is detected at the time of diagnosis in at least half of cases of verrucous leukoplakia, the most common type of OPMD [3]. At the same time, several studies have demonstrated a close relationship between this pathology and infection caused by HPV of high oncogenic risk [1,3]. Oncogenic types, in particular HPV-16 and HPV-18, have been reported to be the most likely causes of carcinogenesis of both the mucous membranes of the cervix, penis, and perianal region, as well as the oral mucosa [1].

The high mortality rate associated with OSCC is the result of late diagnosis of the malignant potential of associated precancerous diseases. Currently, there is no reliable diagnostic tool to accurately identify those lesions that can progress to cancer. The detection of HPV in epithelial dysplasia, according to a number of studies, is a reliable biomarker of pathology and the likelihood of its progression [9].

There are isolated data in the scientific literature on the relationship between immunocompromised patients and the development of precancerous diseases associated with HPV infection [10]. Interferons (IFNs), which have antiviral activity, also have an equally important antiproliferative property. They are able to suppress proliferation, modulate differentiation, apoptosis, and cell migration [11]. It is known that proteins of various HPV genotypes are able to suppress the interferon system, thus disrupting the functioning of the immune system and causing the persistence of the virus [10]. Considering HPV-induced oncogenesis, IFNs can affect the signaling pathways of an infectious agent and, in turn, be affected by it, simultaneously and indirectly carrying out antiproliferative and antiviral reactions [10]. This group of cytokines stimulates the antitumor immune response, contributing to tumor regression or slowing down oncogenesis [11,12]. However, there is evidence that some types of IFNs, in particular type 2 (IFN-γ), in low concentrations can increase the survival of tumor cells and enhance their metastatic potential [12].

Currently, there are no clinical studies proving or disproving the relationship between papillomavirus, interferon status, and manifestations of oral epithelial dysplasia. Thus, all of the above highlights the need to assess the relationship between interferon status and HPV infection in patients with OED and to determine whether these parameters are associated with the severity of epithelial dysplasia.

Our study aimed to evaluate the association between interferon status and human papillomavirus infection in patients with oral epithelial dysplasia. The primary endpoint of our study was the difference in IFN-α/β activity between HPV-positive and HPV-negative patients with OED.

The null hypothesis of our study stated that HPV status and interferon activity are not associated with OED severity.

## 2. Materials and Methods

### 2.1. Study Design

In the period from 2022 to 2026, 180 patients were enrolled in an observational study and were examined at the Department of Surgical Dentistry of the Borovsky Institute of Dentistry, Sechenov First Moscow State Medical University of the Ministry of Health of the Russian Federation (Sechenov University). The study was designed and reported with reference to the STROBE checklist, although some items (e.g., handling of missing data) were limited by the retrospective extraction of clinical records.

The sample size was determined a priori based on the primary comparative objective of the study, namely, to detect a clinically meaningful difference in IFN-α/β biological activity between HPV-positive and HPV-negative patients with OED. A two-sided comparison with a significance level of α = 0.05 and statistical power of 80% was assumed. The expected difference in IFN-α/β activity was defined as a biological activity titer (BAT) of 17.3, with an assumed standard deviation of 40 BAT, corresponding to a standardized effect size (Cohen’s d) of 0.433. These assumptions were based on our pilot sample (a pilot subset of 15 patients). Under these assumptions, the calculated sample size was 84 participants per group. To compensate for potential exclusions, incomplete laboratory measurements, and missing data, the target sample size was increased to 90 participants per group.

Because the primary objective of the study was to investigate the association between HPV status and interferon activity, rather than to estimate the prevalence of HPV in patients with OED, HPV status was considered the principal exposure variable. To ensure adequate statistical power and balanced comparisons between exposure groups, the final analytical cohort consisted of equal numbers of HPV-positive and HPV-negative patients. Patients were enrolled consecutively from the pool of eligible participants until the required sample size for each group was achieved. This sampling strategy was chosen to minimize selection bias while improving the precision of comparative analyses of interferon status.

The inclusion criteria are outlined below:

(1) Age of patients from 18 to 80 years.
(2) Histologically confirmed oral potentially malignant disorders, including leukoplakia (ICD-10: K13.2) and oral lichen planus, with concomitant oral epithelial dysplasia. The diagnosis of OLP was established according to the combined clinicopathological criteria of van der Meij and van der Waal (2003) [13].
(3) Indication for surgical biopsy or excision due to lack of response to conservative therapy; clinical suspicion of disease progression.
(4) Signed written informed consent.

The exclusion criteria are outlined below:

(1) Malignant transformation;
(2) Recent antiviral or immunomodulatory therapy;
(3) Severe systemic disease;
(4) Pregnancy or breastfeeding.

The withdrawal criteria are outlined below:

(1) Exacerbation of concomitant systemic disease during the study;
(2) Participant refusal to continue study participation.

### 2.2. Clinical and Laboratory Assessment

In the preoperative period, interferon status was determined in patients with OPMD by assessing the activity of serum interferon, spontaneous IFN, IFN-α/β, and IFN-γ in the blood. The biological material for the PCR study was obtained by total biopsy of the neoplasm. After a pathohistological study, which revealed signs of OED in patients diagnosed with leukoplakia and OLP, a PCR examination of biopsies for the presence of HPV was performed.

The samples for histological examination were fixed in a 10% formalin solution. Biopsies were embedded in paraffin wax, sections with a thickness of 5 microns were obtained, stained with hematoxylin–eosin, and then studied using standard light microscopy. Histopathological assessment was performed by one experienced pathologist who was blinded to HPV status and interferon results. The pathohistological study was conducted at the Immunogen Pathology Laboratory (Moscow, Russia).

The diagnosis of leukoplakia was established clinically as a white spot or plaque that could not be attributed to any other disease after ruling out lesions associated with mechanical, chemical, or thermal trauma, in accordance with WHO criteria [6]. The diagnosis of OLP was established according to the modified clinicopathological criteria proposed by van der Meij and van der Waal (2003) [13], based on the combined assessment of characteristic clinical and histopathological features. The clinical features included reticular, papular, plaque-like, atrophic, erosive, and bullous variants, particularly the presence of typical bilateral lace-like white streaks. The corresponding histopathological features included a predominantly band-like subepithelial lymphocytic infiltrate, liquefactive degeneration of the basal cell layer, damage to the basement membrane, and the presence of melanophages [13]. In the same biopsy specimens, epithelial dysplasia was assessed independently based on the presence of architectural and cytological abnormalities, including impaired epithelial stratification and maturation, loss of cellular polarity, cellular and nuclear polymorphism, nuclear hyperchromasia and enlargement, an increased nuclear-to-cytoplasmic ratio, abnormal mitotic activity, including mitoses above the basal layer, dyskeratosis, and premature keratinization of individual epithelial cells. The presence and severity of OED were determined by the combination of architectural and cytological changes and their extent throughout the thickness of the epithelial layer according to the WHO classification of head and neck tumors [6], using the three-tiered grading system.

The PCR study was performed at the Laboratory of Molecular Immunology of the Federal State Institution “Research Institute of Vaccines and Sera named after I.I. Mechnikov” of the Russian Academy of Medical Sciences using a kit for DNA isolation from histological preparations fixed in formalin and embedded in neutral paraffin. The HPV KVANT-21 reagent kit (DNA Technology, Moscow, Russia) was used to identify, type, and quantify HPV DNA of low (HPV 6, 11, and 44) and high (HPV 16, 18, 26, 31, 33, 35, 39, 45, 51, 52, 53, 56, 58, 59, 66, 68, 73, and 82) carcinogenic risk. Amplification and detection were performed using a DTprime 5 device (DNA Technology, Moscow, Russia). The diagnostic sensitivity of the HPV KVANT-21 reagent kit is 99.3% (95% CI) and 99.9% (95% CI) specificity. The limit of detection is 5 copies of HPV DNA per amplification tube (or 10^3^ DNA copies/mL). The kit includes a specimen collection control, which is necessary for assessing the threshold value for the number of epithelial cells in the sample: the quality criterion is a value greater than 10^4^ EC/sample; otherwise, the material was considered insufficient. The assay included positive and negative controls in each run, and HPV positivity was interpreted according to the manufacturer’s validated criteria for target amplification and genotype identification.

The biological activity of interferons (IFN status) was determined in the interferon laboratory of the Federal State Institution “Research Institute of Vaccines and Sera named after I.I. Mechnikov” of the Russian Academy of Medical Sciences in vitro with a micromethod according to the developed author’s methodology [14]. During the experiments, whole heparinized blood of patients was used. The following indicators of IFN activity in vitro were evaluated: circulating (serum) IFN; type I IFN (α/β), type II IFN (γ), and spontaneous IFN. The technique makes it possible to assess the degree of insufficiency of the interferon system in terms of the ability to produce biologically active interferon by blood leukocytes, which is expressed in biological activity titers. The normal values of the bioactivity of type I IFN were BAT ≥ 640, type II IFN ≥ 64, and serum and spontaneously produced IFN < 2–8 and <2 BAT, respectively. The choice of this research method is justified by the fact that it is a functional test that determines not only the concentration of interferons but also their biological activity, thereby assessing the functional state of the interferon system.

### 2.3. Ethical Considerations

The study was conducted in accordance with the Declaration of Helsinki and approved by the ethics committee (Protocol No. 25-22, dated 8 December 2022). Written informed consent was obtained from all participants.

### 2.4. Statistics

All collected data were organized into statistical tables, followed by statistical processing and analysis using IBM SPSS Statistics version 28.0 and R statistical software version 4.0.5. The primary comparative outcome was the difference in IFN-α/β activity between HPV-positive and HPV-negative patients. Secondary outcomes included differences in IFN-γ activity according to HPV status, the association between interferon activity and OED grade, and the association between HPV status and OED severity. For secondary clinical analyses, OED severity was dichotomized into severe versus mild/moderate disease.

A descriptive method for calculating averages, standard deviations (SDs), medians, and minimum (min)–maximum (max) numbers was used to characterize the quantitative data. Pearson’s chi-square test was used to compare categorical variables. The strength of statistically significant associations was quantified using Cramer’s V effect size. The normality of the sample distribution was assessed using the Kolmogorov–Smirnov test. The Mann–Whitney test was used when comparing groups with respect to quantitative indicators with an abnormal distribution. For three-group comparisons of interferon activity across OED severity categories (mild/moderate/severe), the Kruskal–Wallis test was used, followed by post hoc pairwise Dunn’s tests with Holm correction for multiple comparisons. The Spearman correlation coefficient (Spearman’s rho) was used to assess the strength and direction of a monotonic association between two variables. The results were considered statistically significant with a confidence level of at least 95% (*p* < 0.05). As an additional exploratory analysis, patients were stratified into HPV-negative, low-risk HPV only, and high-risk HPV with or without low-risk HPV groups. OED grade distribution was compared using Pearson’s chi-square test, with Cramer’s V reported as an effect size measure.

A multivariate binary logistic regression analysis was performed to identify factors independently associated with severe OED. Severe OED was considered the dependent variable (severe vs. mild/moderate). The independent variables included HPV status, IFN-α/β activity, IFN-γ activity, age, sex, and clinical diagnosis (leukoplakia vs. oral lichen planus). The variables included in the model were selected a priori based on the study objectives and previously published evidence regarding the associations of HPV infection, OED severity, and interferon-mediated immune responses [1,3,10,11,12]. All variables were entered simultaneously using the forced-entry method. Multicollinearity was assessed using variance inflation factors (VIFs), all below 2.0. HPV status and interferon parameters were selected as the primary variables of interest, while age, sex, and clinical diagnosis were included as potential confounding factors. Regression coefficients (β), adjusted odds ratios (ORs), 95% confidence intervals (95% CIs), and *p*-values were calculated for each variable. IFN activities were scaled per 10 BAT units to facilitate clinical interpretation. Model calibration was assessed using the Hosmer–Lemeshow goodness-of-fit test. Receiver operating characteristic (ROC) curve analysis was performed to evaluate the diagnostic performance. The area under the ROC curve (AUC), sensitivity, specificity, and the optimal probability threshold determined by the Youden index were calculated.

IFN activity was assessed twice by two immunologists. Reproducibility was assessed using a two-way random-effects intraclass correlation coefficient (ICC) for absolute agreement and single measurements, ICC (2,1), with 95% CIs. The ICC was calculated separately for IFN-α/β and IFN-γ and separately in HPV-positive and HPV-negative patients, as well as in the pooled sample. CIs were estimated using nonparametric bootstrap resampling with 20,000 repetitions. ICC values were interpreted according to the criteria proposed by Koo TK et al. [15]. The final values used in the clinical analyses were calculated by averaging the measurements obtained by the two immunologists.

## 3. Results

Based on the inclusion and exclusion criteria, the study included an equal number of HPV-positive (*n* = 90; 50%) and HPV-negative (*n* = 90; 50%) patients aged 18 to 80 years with diagnoses of “Leukoplakia and other changes in the oral mucosa” (K 13.2) and oral lichen planus with signs of epithelial dysplasia.

Baseline demographic characteristics are presented in Table 1. No statistically significant differences were identified between the groups in terms of age or sex distribution (*p* > 0.05).

Of all the patients examined who were included in the study, 66 (36.7%) were found to have severe OED, 97 (53.9%) had moderate OED, and 17 (9.4%) had mild OED. Among the patients examined, 103 were diagnosed with leukoplakia and 77 with OLP. The distribution of HPV status and OED severity according to the clinical diagnosis is presented in Table 2. HPV positivity was more frequent among patients with leukoplakia than among those with OLP; however, this difference was not statistically significant (Pearson’s χ^2^ = 0.57, *p* = 0.451). We also found that the overall distribution of OED severity did not differ significantly between the two diagnostic groups (Pearson’s χ^2^ = 5.17, *p* = 0.076) (Table 2).

A significant association was observed between HPV status and the severity of OED (Pearson’s χ^2^ = 7.6, df = 2, *p* = 0.022), with a small-to-moderate effect size (Cramer’s V = 0.205). Severe OED was identified in 45.6% (41/90) of HPV-positive patients compared with 27.8% (25/90) of HPV-negative patients, whereas mild lesions were more frequently observed in HPV-negative individuals (13.3% vs. 5.6%, respectively).

When assessing the prevalence of individual papillomavirus strains among HPV-positive patients with dysplastic lesions of the oral mucosal epithelium, we detected HPV type 6—which belongs to the low-oncogenic risk group—in the majority of cases (*n* = 46; 51.1%) (Table 3). Several HPV genotypes were detected simultaneously in some patients; therefore, the total percentage exceeds 100%. Percentages were calculated based on the total number of HPV-positive patients (*n* = 90).

To further explore the potential differences between HPV risk categories, HPV-positive patients were classified into those with low-risk HPV infection only and those with high-risk HPV infection, with or without concomitant low-risk HPV types (“high-risk HPV ± low-risk HPV”) (Table 4). Severe OED was observed in 27.8% of HPV-negative patients, 48.3% of patients with low-risk HPV infection only, and 44.3% of patients with high-risk HPV infection with or without low-risk HPV co-infection. The overall association between HPV risk category and OED grade was not statistically significant (Pearson’s χ^2^ = 7.895, df = 4, *p* = 0.096; Cramer’s V = 0.148). When high-risk HPV status was specifically compared with HPV-negative status across OED grades, a trend toward a higher proportion of severe OED was observed among patients with high-risk HPV infection (44.3% vs. 27.8%), although the association did not reach statistical significance (χ^2^ = 5.886, df = 2, *p* = 0.053; Cramer’s V = 0.197).

Based on the results of molecular genetics and immunological studies, statistically significant differences were identified in the assessment of interferon status between HPV-positive and HPV-negative patients. Significantly reduced activity of both IFN-α/β and IFN-γ was observed in patients with detected papillomavirus (Table 5). No differences in serum and spontaneously produced IFN levels were observed between the study groups.

Reproducibility of IFN-α/β and IFN-γ measurements was assessed using a two-way random-effects ICC for absolute agreement and single measurements, ICC (2,1). Across all four measurements obtained for each patient (two attempts by each of two immunologists), agreement was excellent. In the HPV-positive group, the ICC was 0.998 (95% CI, 0.993–1.000) for IFN-α/β and 1.000 (95% CI, 1.000–1.000) for IFN-γ. In the HPV-negative group, the ICC was 1.000 (95% CI, 1.000–1.000) for IFN-α/β and 0.995 (95% CI, 0.981–1.000) for IFN-γ. In the pooled sample, the ICC was 0.999 (95% CI, 0.997–1.000) for IFN-α/β and 0.998 (95% CI, 0.995–1.000) for IFN-γ.

Interferon activity decreased with increasing OED severity. The largest separation was observed for IFN-α/β (*p* < 0.001), where the median BAT value was 40 in severe OED and 160 in mild OED, indicating a 120 BAT-unit difference. A smaller but significant reduction was also found for IFN-γ (*p* < 0.05), with median BAT values of 12 and 16, respectively (Table 6).

A correlation analysis revealed an association between IFN-γ and IFN-α/β in a group of patients with moderate OED: Spearman’s rho = 0.295 (*p* = 0.0033).

Multivariate binary logistic regression analysis identified HPV positivity and reduced IFN-α/β activity as factors independently associated with severe OED (Table 7). HPV-positive patients had higher odds of severe OED compared with HPV-negative individuals (adjusted OR = 4.62, 95% CI: 2.18–9.79, *p* < 0.001). In addition, each 10-unit decrease in IFN-α/β activity was associated with a 34% increase in the odds of severe OED (adjusted OR = 1.34, 95% CI: 1.14–1.58, *p* < 0.001). Conversely, each 10-unit increase in IFN-α/β activity was associated with lower odds of severe OED (adjusted OR = 0.75, 95% CI: 0.63–0.88, *p* < 0.001). After adjustment for potential confounders, IFN-γ activity, age, sex, and clinical diagnosis were not independently associated with dysplasia severity. The final model demonstrated good calibration (Hosmer–Lemeshow *p* = 0.58) and moderate explanatory power (Nagelkerke R^2^ = 0.36).

ROC curve analysis was performed to evaluate the discriminatory ability of the multivariable logistic regression model for identifying patients with severe OED (Figure 1).

The combined model demonstrated acceptable discriminatory ability (AUC = 0.736). The optimal probability threshold determined by the Youden index was 0.282, yielding a sensitivity of 89.4% and a specificity of 56.1% for the detection of severe OED. The combined model showed moderate discriminatory ability with high sensitivity but modest specificity. A combined assessment of virological and immunological parameters demonstrated an association with the presence of severe OED.

## 4. Discussion

The present study investigated the relationship between HPV infection, interferon status, and the severity of oral epithelial dysplasia in patients with oral potentially malignant disorders. The present study demonstrated a significant association between HPV infection, reduced interferon activity, and increasing severity of OED, suggesting that impaired antiviral immunity may be associated with greater OED severity.

Although the role of HPV has been extensively studied in cervical and anogenital neoplasia, evidence regarding OED remains limited. Sillman et al. [16] first demonstrated that impaired immunity contributes to the persistence and progression of HPV-associated epithelial lesions. Our findings extend these observations to OPMD, supporting the hypothesis that reduced antiviral immunity may be associated with HPV positivity and higher dysplasia grade.

A key finding of this study is the significantly reduced activity of both IFN-α/β and IFN-γ in HPV-positive patients. Interferons play a central role in antiviral immunity, whereas HPV oncoproteins E6 and E7 are known to inhibit interferon signaling and promote immune evasion [10,11,17]. Recent reviews further indicate that persistent HPV infection promotes immune evasion by suppressing interferon-stimulated genes, impairing antigen presentation, and remodeling the local immune microenvironment, thereby facilitating viral persistence and epithelial transformation [18,19,20]. The observed reduction in interferon activity is consistent with these mechanisms. Growing evidence suggests that disruption of interferon signaling contributes to immune escape and early carcinogenic events in HPV-associated oral lesions, supporting the biological relevance of our findings [21,22]. Furthermore, patients with severe dysplasia exhibited the lowest interferon activity, suggesting that impaired interferon-mediated immune surveillance may be associated with more severe dysplasia [10,11].

In the multivariate model, lower IFN-α/β activity was independently associated with higher odds of severe OED after adjustment for HPV status, age, sex, and clinical diagnosis. Each 10-unit increase in IFN-α/β activity was associated with a 25% reduction in the odds of severe OED (adjusted OR = 0.75, 95% CI: 0.63–0.88; *p* < 0.001), corresponding to a 34% increase in odds for each 10-unit decrease. This finding suggests that reduced type I interferon activity may be independently associated with greater dysplasia severity. Given the pivotal role of type I interferons in antiviral innate immunity, decreased IFN-α/β activity may reflect persistent immune dysregulation associated with the severity of OED.

The role of IFN-γ in carcinogenesis remains complex. While IFN-γ may exert both tumor-promoting and tumor-suppressive effects depending on the biological context [12], substantial evidence supports its protective function during early carcinogenesis through enhancement of antigen presentation, activation of cytotoxic immune responses, and induction of apoptosis [23]. Accordingly, the reduced IFN-γ activity observed in severe lesions may reflect weakening of antitumor immune defense associated with greater dysplasia severity. This interpretation is also consistent with recent evidence indicating that defective antiviral immunity favors persistent HPV infection and increases cancer susceptibility, particularly in immunocompromised settings [24].

Another noteworthy finding was the predominance of HPV-6 among HPV-positive lesions, with this genotype detected in 51.1% of HPV-positive patients. Previous studies have mainly associated oral dysplasia with high-risk genotypes, particularly HPV-16 and HPV-18 [1,8,9]. However, our findings are consistent with recent reports demonstrating considerable variability in HPV genotype distribution among OPMD [25]. Although HPV-6 is generally classified as a low-risk genotype, occasional reports of its association with severe dysplastic epithelial lesions suggest that its biological significance may be underestimated [26]. To further explore whether HPV oncogenic risk was related to the severity of epithelial dysplasia in our cohort, we additionally stratified patients into HPV-negative, low-risk HPV infection only, and high-risk HPV infection with or without concomitant low-risk HPV infection. No significant difference in OED grade distribution was observed between patients with high-risk HPV infection and those with low-risk HPV infection only. These findings suggest that, within the present cohort, the HPV oncogenic risk category alone may not fully explain the observed differences in dysplasia severity. The predominance of HPV-6 may reflect geographic variation, differences in study populations, or methodological differences in HPV detection, as similar heterogeneity has been reported in epidemiological studies of OPMD [25,26]. Nevertheless, the high prevalence of HPV-6 warrants further investigation, particularly in larger cohorts with assessment of viral persistence, viral load, and genotype-specific associations with epithelial dysplasia.

From a clinical perspective, the combined assessment of HPV status and interferon activity may provide complementary information for identifying patients with more severe OED in addition to conventional histopathological grading [2,3,5]. Such biomarkers may potentially complement histopathological assessment when characterizing patients with severe dysplastic lesions. Recent studies have emphasized that combining molecular and immunological biomarkers with conventional histopathological assessment may improve the characterization and stratification of HPV-associated lesions [27].

Overall, the present findings indicate that impaired interferon-mediated immunity is closely associated with HPV infection and increasing dysplasia severity. The observed association highlights the potential value of integrating virological and immunological biomarkers into future studies of OED severity.

### Limitations and Future Directions

Several limitations should be considered when interpreting these findings. First, the retrospective extraction of some clinical variables (including tobacco and alcohol use) precluded their inclusion in the multivariable model, which may have led to residual confounding, particularly for leukoplakia cases where these exposures are well-established risk factors. Second, although interferon activity was assessed using repeated measurements by two immunologists, measurements were obtained at a single time point for each patient; therefore, temporal fluctuations in interferon status could not be evaluated. Third, the single-center design and the relatively wide age range (18–80 years) may limit generalizability to other populations with different HPV epidemiology or genetic backgrounds. Fourth, the study was conducted at a single clinical center, which may limit the generalizability of the findings to other populations.

Finally, an important methodological limitation concerns the diagnostic criteria used for OLP in patients with concomitant OED. In the present study, OLP was diagnosed according to the modified clinicopathological criteria of van der Meij and van der Waal (2003) [13], based on characteristic clinical and histopathological features, while epithelial dysplasia was assessed separately according to the WHO three-tier classification. More contemporary criteria have been proposed by the American Academy of Oral and Maxillofacial Pathology [28], which include the absence of epithelial dysplasia among the histopathological criteria supporting an OLP diagnosis. Therefore, their application could potentially result in reclassification of some cases with concomitant OED and should be considered when interpreting the OLP subgroup.

Future research should address these limitations through prospective, multicenter studies with larger and more diverse cohorts. Longitudinal designs with repeated measurements of interferon status and other immune parameters would enable assessment of temporal dynamics and may help determine whether reduced interferon activity precedes changes in dysplasia severity or is a consequence of more advanced disease. Systematic collection of detailed data on tobacco and alcohol use, as well as other potential confounders (e.g., occupational exposures, comorbidities, and medication use), is needed to better isolate the independent contribution of HPV and interferon status to dysplasia severity. Additional studies should also explore the biological mechanisms linking HPV persistence, interferon signaling, and epithelial transformation, including local mucosal immunity, viral load, and genotype-specific effects. Finally, prospective longitudinal studies and external validation in independent cohorts are required to determine whether the observed associations have implications for subsequent changes in dysplasia severity or malignant transformation.

## 5. Conclusions

The results demonstrate a correlation between manifestations of OED, HPV infection, and the patients’ interferon status. Interferon status indicators in patients with oral dysplastic lesions associated with HPV infection were significantly lower compared to the HPV-negative group. Furthermore, severe OED was associated with lower activity of IFN-α/β than moderate and mild OED. Thus, suppression of IFN-α/β and IFN-γ activity in HPV-positive patients may reflect an association with more severe dysplastic changes. Assessment of interferon status, together with HPV detection, may serve as a valuable adjunctive approach for identifying patients with severe dysplastic changes, warranting further validation in larger prospective studies.

## Figures and Tables

**Figure 1:**
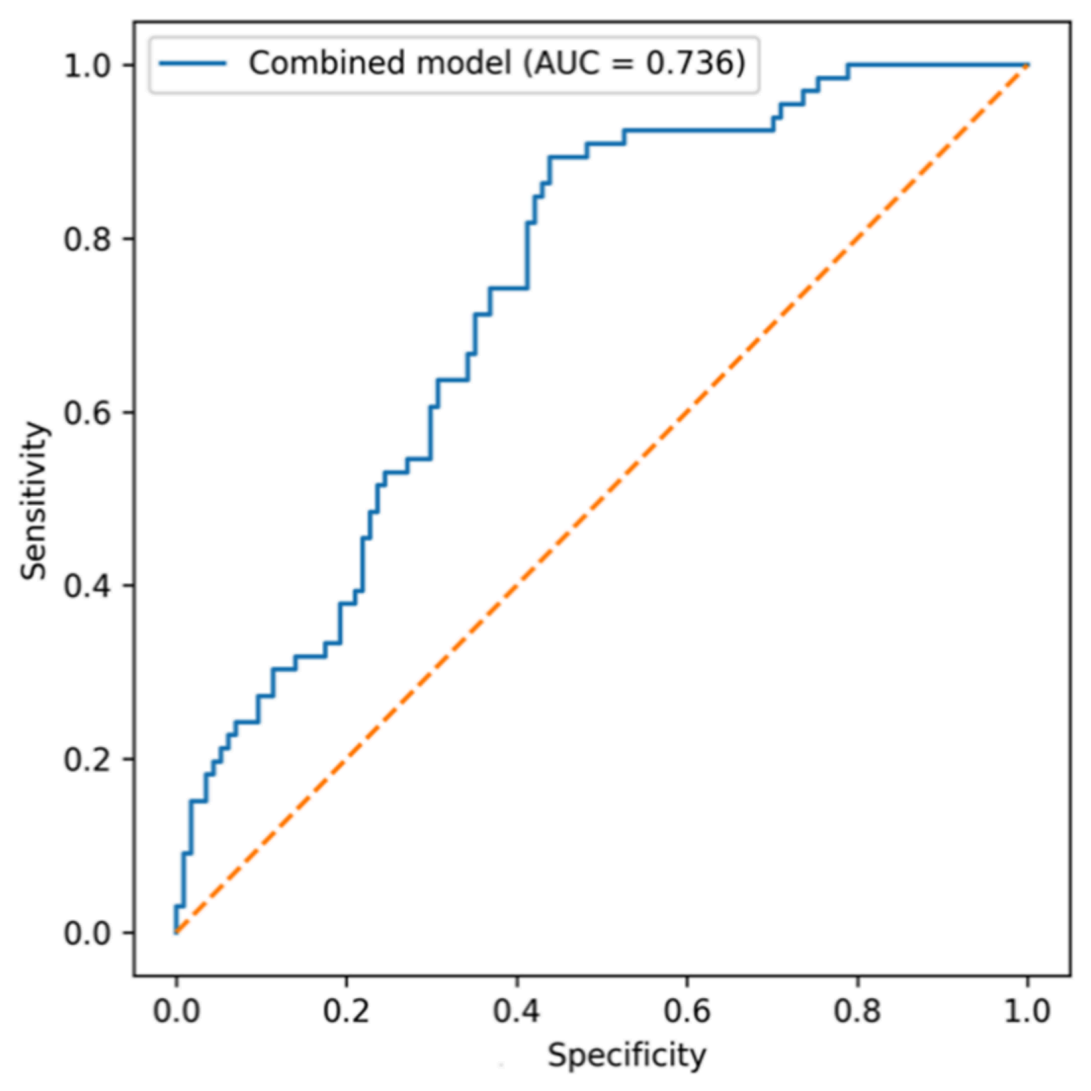
ROC curve of the combined multivariable model for severe OED.

**Table 1:** Patient demographics.

Variables	HPV-Positive Patients	HPV-Negative Patients
Age, years (mean ± SD)	53.24 ± 17.24	47.63 ± 15.96
Median	56	52
Minimum	19	21
Maximum	78	67
Sex (*n* %)		
men	46.50%	43.20%
women	53.50%	56.80%

**Table 2:** Distribution of clinical diagnosis, HPV status, and OED severity.

Diagnosis	HPV-Positive *n* (%)	HPV-Negative *n* (%)	Severe OED	Moderate OED	Mild OED	Total
Leukoplakia	54 (52%)	49 (48%)	44 (43%)	48 (47%)	11 (11%)	103
OLP	36 (47%)	41 (53%)	22 (29%)	49 (63%)	6 (8%)	77
Total	90	90	66	97	17	180

**Table 3:** Distribution of HPV genotypes in HPV-positive patients with OED.

HPV Genotype	*n*	% of HPV-Positive Cases
HPV-6	46	51.1
HPV-11	14	15.6
HPV-16	39	43.3
HPV-18	24	26.7
HPV-31	20	22.2
HPV-33	9	10.0
HPV-45	17	18.8
HPV-59	2	2.2
HPV-82	4	4.4

**Table 4:** Distribution of OED grades according to HPV risk category.

HPV STATUS	Severe OED, *n* (%)	Moderate OED, *n* (%)	Mild OED, *n* (%)	Total
HPV-negative	25 (27.8)	53 (58.9)	12 (13.3)	90
Low-risk HPV only	14 (48.3)	13 (44.8)	2 (6.9)	29
High-risk HPV ± low-risk HPV	27 (44.3)	31 (50.8)	3 (4.9)	61
Total	66 (36.7)	97 (53.9)	17 (9.4)	180

**Table 5:** IFN-α/β and IFN-γ activity by HPV status.

Biological Activity	HPV-Positive Mean ± SD (Median; Min–Max)	Biological Activity	HPV-Negative Mean ± SD (Median; Min–Max)	*p* Value (Mann- Whitney)
IFN-α/β	43.11 ± 29.89 (40; 10–160)	IFN-α/β	86.22 ± 48.50 (80; 40–240)	<0.001
IFN-γ	11.33 ± 2.95 (12; 8–16)	IFN-γ	13.42 ± 2.90 (14; 8–16)	<0.001

SD—standard deviation.

**Table 6:** Interferon status of patients based on the severity of OED.

Biological Activity	Severe OED Mean ± SD (Median; Min–Max)	Biological Activity	Moderate OED Mean ± SD (Median; Min–Max)	Biological Activity	Mild OED Mean ± SD (Median; Min–Max)	*p* Value (Kruskal- Wallis)	Post-Hoc Pairwise Comparisons (Dunn’s Test; Holm-Adjusted *p*)
IFN-α/β	51.21 ± 30.46 (40; 10–160)	IFN-α/β	73.09 ± 53.08 (60; 10–240)	IFN-α/β	141.18 ± 51.22 (160; 80–240)	<0.001	Severe vs. moderate: *p* = 0.0065 Severe vs. mild: *p* < 0.001 Moderate vs. mild: *p* < 0.001
IFN-γ	12.36 ± 3.04 (12; 8–16)	IFN-γ	12.41 ± 3.03 (12; 8–16)	IFN-γ	14.59 ± 1.97 (16; 12–16)	0.016	Severe vs. moderate: *p* = 0.9191 (ns) Severe vs. mild: *p* = 0.0178 Moderate vs. mild: *p* = 0.0178

ns—not significant; SD—standard deviation.

**Table 7:** Multivariate binary logistic regression analysis of factors independently associated with severe OED.

Variable	β Coefficient	Adjusted OR	95% CI	*p*-Value
HPV-positive	1.53	4.62	2.18–9.79	<0.001
IFN-α/β activity (per 10 BAT increase)	−0.29	0.75	0.63–0.88	<0.001
IFN-γ activity (BAT)	0.11	1.12	0.94–1.34	0.182
Age	0.02	1.02	0.99–1.05	0.118
Female sex	0.14	1.15	0.61–2.18	0.664
Oral lichen planus	0.27	1.31	0.69–2.47	0.407

## Data Availability

The raw data supporting the conclusions of this article will be made available by the authors upon request.

## Abbreviations

The following abbreviations are used in this manuscript:

OPMDs	Oral potentially malignant diseases
HPV	Human papillomavirus
OED	Oral epithelial dysplasia
IFN	Interferon
OSCC	Oral squamous cell carcinoma
OLP	Oral lichen planus
VIFs	Variance inflation factors
ROC	Receiver operating characteristic
AUC	Area under the ROC curve
ICC	Intraclass correlation coefficient
